# Capacitance-Based Biosensor for the Measurement of Total Loss of L-Amino Acids in Human Serum during Hemodialysis

**DOI:** 10.1021/acssensors.2c01342

**Published:** 2022-10-21

**Authors:** Justas Miškinis, Eimantas Ramonas, Vidutė Gurevičienė, Julija Razumienė, Marius Dagys, Dalius Ratautas

**Affiliations:** Life Science Center, Vilnius University, Saulėtekio al. 7, Vilnius LT-10257, Lithuania

**Keywords:** capacitance-based biosensor, L-amino acid oxidase, L-amino acids, hydrogen peroxide, human serum

## Abstract

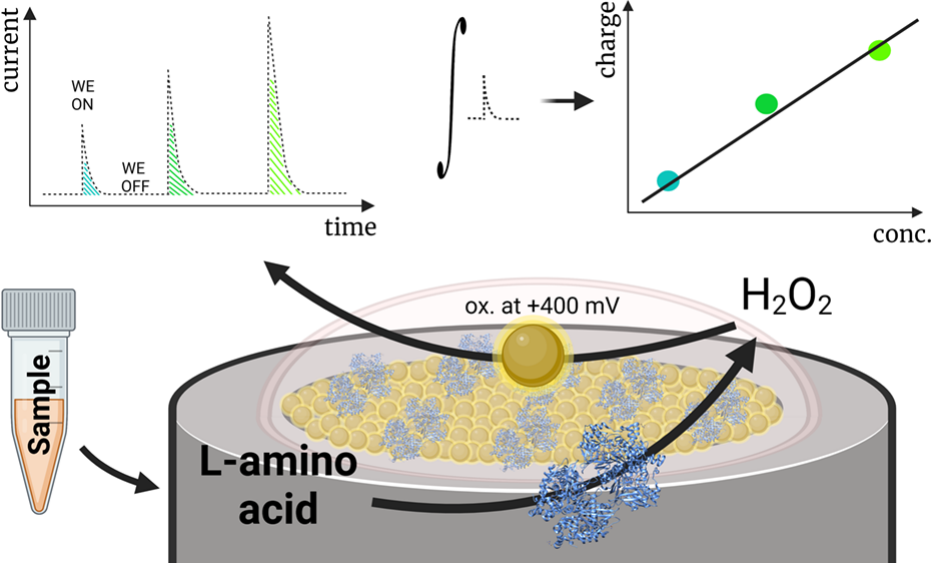

In this paper, we present a biosensor based on a gold nanoparticle (AuNP)-modified Pt electrode with an adjusted membrane containing cross-linked L-amino acid oxidase for the detection and quantification of total L-amino acids. The designed biosensor was tested and characterized using the capacitance-based principle, capacitance measurements after electrode polarization, disconnection from the circuit, and addition of the respective amount of the analyte. The method was implemented using the capacitive and catalytic properties of the Pt/AuNP electrode; nanostructures were able to store electric charge while at the same time catalyzing the oxidation of the redox reaction intermediate H_2_O_2_. In this way, the Pt/AuNP layer was charged after the addition of analytes, allowing for much more accurate measurements for samples with low amino acid concentrations. The combined biosensor electrode with the capacitance-based measurement method resulted in high sensitivity and a low limit of detection (LOD) for hydrogen peroxide (4.15 μC/μM and 0.86 μM, respectively) and high sensitivity, a low LOD, and a wide linear range for L-amino acids (0.73 μC/μM, 5.5 μM and 25–1500 μM, respectively). The designed biosensor was applied to measure the relative loss of amino acids in patients undergoing renal replacement therapy by analyzing amino acid levels in diluted serum samples before and after entering/leaving the hemodialysis apparatus. In general, the designed biosensor in conjunction with the proposed capacitance-based method was clinically tested and could also be applied for the detection of other analytes using analyte-specific oxidases.

The determination of free L-amino acids in human biological fluids (blood, serum, etc.) is an important parameter associated with the function of various organs, some cancers, human metabolism, and various inflammatory or neurological diseases.^1−5^ Recently, the importance of monitoring general levels of L-amino acids in patients undergoing renal replacement therapy was reported, demonstrating that patients could lose up to 5–15 g/day of L-amino acids.^6,7^ However, the clinical implementation of L-amino acid analysis in biological fluids is lacking mainly because of the complexity of currently available methods such as high-performance liquid chromatography which typically are costly and time-consuming.^8−10^

Electrochemical (bio)sensors could be an attractive solution for routine measurements of L-amino acids, as these devices were successfully engineered and applied for routine measurements of various clinically relevant analytes, for example, glucose,^11,12^ formaldehyde,^13^ glycerol,^14^ lactate,^15^ and so on.^16^ Yet, to the best of our knowledge, no biosensors, which could be applicable to the analysis of real clinical samples for total L-amino acid determination, were reported in the literature. Most of the recent work focused on the determination of some L-amino acids, rather than the determination of the total concentration of the main L-amino acids. For example, Nanjo and Guilbault reported on one of the first studies toward the development of biosensors for the determination of L-amino acids.^17^ In their work, an enzyme electrode for the detection of L-amino acids was designed based on immobilized L-amino acid oxidase for the oxidation of L-amino acids. However, the sensor was suitable for the determination of only several amino acids and was not tested using clinical samples. Several other biosensors have also been developed: Kwan et al. have developed biosensors based on L-amino acid oxidase and protease and have demonstrated analysis of some L-amino acids and even peptides of economic interest^18^ and later reported an amperometric biosensor for the determination of L-alanine in various beverages (such as sport drinks);^19^ Váradi et al., reported an amperometric detection system suitable for the differentiation between D- and L-amino acids based on immobilized L-/D-amino acid oxidases;^20^ Sarkar et al., demonstrated amperometric biosensors with L-/D-amino acid oxidases for general purpose measurement of L-/D-amino acids and applied those sensors to measure the effects of milk aging.^21^ More biosensors were also developed for the determination of specific individual amino acids; that is, according to a recent review by Moulaee and Neri most papers published on electrochemical biosensing of amino acids involve the detection of cysteine (47%), tryptophan (22%), and tyrosine (18%).^22^ In clinically relevant samples (blood or serum), the concentrations of total L-amino acids generally are higher compared to those of individual amino acids (0.5–6 mM vs 0–0.45 mM).^23^ However, the measurements are complex because many L-amino acids should be evaluated during the same measurement from the same sample volume, and real samples usually give significant interference at a low dilution ratio (5–10 times), while a high dilution ratio (10–100) typically removes the interference but makes the concentrations too low for the accurate measurements for conventional amperometric or potentiometric methods. As a result, most likely, the above discussed problems limited the biosensor development for the analysis of general L-amino acids in real clinical samples.

In recent years, a few groups have been working toward an elegant solution to increase the sensitivity of various sensors by means of improving the measurement method of conventional amperometry or potentiometry to include a capacitance-based element. The most notable studies come from the research groups of Bobacka^24,25^ and Bakker.^26−28^ For example, in a study by Hupa et al., a new signal transduction principle for solid-contact ion-selective electrodes was introduced and named constant-potential coulometry.^24^ The idea behind the method was to measure current over time at constant potential, while changing the concentration of an analyte K^+^. The change in analyte concentrations resulted in current jumps, which in turn were calculated to give certain capacitance values with a correlation to the concentration of analyte.^25^ Another recent study utilizing a similar principle was reported by Kraikaew et al. where a capacitive readout was utilized to measure very small changes in pH and applied for the seawater measurements.^27^ The idea behind the method proposed by Kraikaew et al. was to incorporate a capacitor in series to the pH probe while maintaining a constant potential. As a result, even very low changes in pH (e. g., Δ0.001 pH) triggered current changes stored in a capacitor resulting in a linear relationship between ΔpH and the capacitance.

In our work, we demonstrate a biosensor for the detection and quantification of total L-amino acids utilizing a similar capacitance-based method. The biosensor was based on a gold nanoparticle (AuNP)-modified platinum (Pt) electrode with a surface-attached membrane containing cross-linked L-amino acid oxidase. The designed biosensor was tested and characterized using the capacitance-based method: measurements were recorded after the electrode polarization, disconnection from the circuit, and addition of analytes. Charge accumulation was achieved using the capacitive and catalytic properties of the Pt/AuNP layer: the electrode was able to catalyze the oxidation of the enzymatic reaction product H_2_O_2_ and store the received electric charge within the capacitive layer. The combined biosensor electrode with the capacitance measurement method resulted in high sensitivity, a low limit of detection (LOD) for hydrogen peroxide (4.15 μC/μM and 0.86 μM, respectively) and high sensitivity, a low LOD, and a wide linear range for L-amino acids (0.73 μC/μM, 5.5 μM and 25–1500 μM, respectively). Furthermore, the enzymatic electrode demonstrated adequate stability and retained approximately 50% of the initial activity after 10 days of storage. Finally, the designed biosensor was tested to measure amino acid concentrations in multiple diluted human serum samples taken from patients undergoing renal replacement therapy. Key novelty points of our work compared to previous work are as follows: (i) the capacitive-based method can be further expanded in use for not only ion detection but also clinically relevant compounds such as L-amino acids; (ii) the method can be applied to create enzymatic biosensors; and (iii) the designed enzymatic biosensors utilizing the capacitance-based method can be successfully utilized to measure analytes in clinically relevant samples (diluted human serum).

## Experimental Section

### Materials

Gold(III) chloride trihydrate, sodium citrate, NaH_2_PO_4_·2H_2_O, KCl, and amino acid standard were purchased from Sigma-Aldrich. L-Amino acid oxidase from*Crotalus adamanteus* (LAOx) was purchased from Sigma-Aldrich. All experiments were carried out using working buffer solution (WBS) containing 50 mM NaH_2_PO_4_·2H_2_O and 100 mM NaCl, and the pH was adjusted to 7.2 using HCl. AuNPs were synthesized using HAuCl_4_·3H_2_O and trisodium citrate according to the revised Turkevich synthesis method.^29^ Subsequently, AuNPs were concentrated by centrifugation (12,000 rpm, 15 min). Around 90% of the supernatant was removed; the remaining dispersion was collected in a new test tube. The diameter of AuNPs and the concentration of the prepared stock solution were determined to be 18 nm and 0.337 μM using the spectrophotometric method.^30^

The enzyme membrane containing LAOx was prepared using a semipermeable PET film (thickness 12 μm, pore diameter 0.4 μm) as a base purchased from Joint Institute of Nuclear Research (Russia). The enzymatic membrane was constructed by mechanically attaching and fixing the multilayer membrane containing immobilized LAOx to the surface of the working electrode. As a result, LAOx was covalently immobilized on the flexible PET support using albumin and glutaraldehyde and was sufficiently stable.

### Electrode Preparation Procedures

The platinum electrodes were polished using a fine-grit pad surface, obtained from BASi, and wetted with deionized water. The electrodes were thoroughly rinsed with deionized water and dried. The deposition of AuNPs was carried out on the surface of the platinum electrodes placing 5.0 μL of colloidal AuNP solution and allowing it to dry at room temperature. Once the surface was dried, the electrodes were rinsed with deionized water and dried by blowing argon gas. Electrodes prepared according to these procedures are further named Pt/AuNP. Subsequently, the enzymatic membrane was placed tightly on the electrode to fully cover the active surface. Electrodes prepared accordingly are further named Pt/AuNP/Enz.

### Methods and Measurements

Electrochemical experiments were performed with a low-current potentiostatic system from UAB “Bioanalizės sistemos”, Lithuania, capable of sampling working electrode current output as fast as 15,000 reads per second at 24-bit resolution. Amperometric, coulometric, and cyclic voltammetry measurements were performed in a three-electrode glass cell using a silver chloride electrode (Ag/AgCl, 205 mV vs SHE) as a reference electrode. The titanium electrode (surface area 1.65 cm^2^) was used as the counter electrode, and the platinum electrode (surface area 0.057 cm^2^) was used for the construction of biosensor electrodes. All the working electrode potential values referred in this study are reported as *versus* silver chloride electrode used.

Capacitance measurements were conducted by using multistep amperometry. The measurement algorithm consisted of potential-current steps. At first, the electrode was polarized at 400 mV without the analyte (H_2_O_2_ or L-amino acids) allowing the electrode surface to fully discharge. Subsequently, the electrode was disconnected from the circuit and thus left polarized at residual potential of about the same 400 mV value after the addition of the analytes for 100 s. Once the analytes were added, the discharged electrode oxidized the analytes and accumulated the electric charge. Subsequently, the electrode was connected to a circuit, and the potential-current step was conducted once again to polarize the electrode to 400 mV. The current flowing to the electrode was recorded for 2000 ms and was used to calculate the total electrode charge.

### Human Serum Samples

Human serum samples from patients undergoing renal replacement therapy were received from Vilnius university hospital Santaros clinics with the approval of the Vilnius Regional Biomedical Research Ethics Committee (approval number: 2021/2-1306-784). Human serum samples were measured using capacitance-based biosensors as received without any additional modifications. The dilution of the samples was 11, that is, 100 μL of the serum sample was placed in a measurement cell containing 1000 μL of WBS. All samples were measured in parallel with the alternative colorimetric method to verify the validity of the designed biosensor electrodes. Before measuring the samples using the colorimetric method, the samples were diluted five times by mixing 20 μL of serum with 80 μL of WBS as prepared as described. The diluted samples were thermally inactivated by heating at 90 °C for 15 min.^31^ After thermal inactivation, the samples were left to reach room temperature and centrifuged for 15 min at 1500 × *g* to remove the precipitate. The concentration of L-amino acids was measured in a received supernatant according to the supplier’s technical bulletin (Sigma-Aldrich product code: MAK002-1KT). Briefly, 50 μL of supernatant was placed in the well of a 96-well plate and mixed with 50 μL of the master mix (composed of working buffer solution, enzyme mixture, and a probe) and incubated at 37 °C for 30 min. After incubation, the absorbance at 570 nm wavelength was measured, compared to the blank, and the concentration of L-amino acids was calculated according to the calibration curve and adjusted according to the sample dilution. The calibration curve was also obtained by mixing 50 μL of standard amino acid solutions (0, 0.16, 0.32, 0.48, 0.64, 0.8 mM) with 50 μL of the master mix.

## Results and Discussion

### Electrochemical Analysis of the Pt/AuNP Electrode

**Figure 1:**
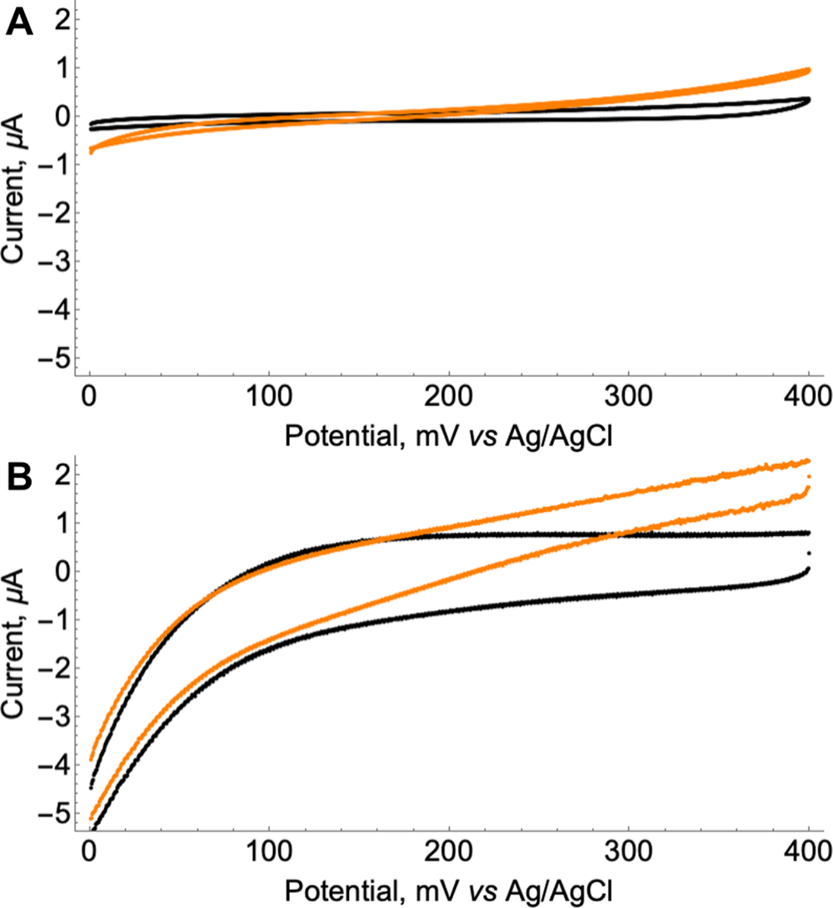
Electrochemical analysis of Pt and Pt/AuNP electrodes. (A) CVs of Pt electrodes without (black curve) and with 1.0 mM H_2_O_2_ (orange curve). (B) CVs of Pt/AuNP electrodes without (black curve) and with 1.0 mM H_2_O_2_ (orange curve). WBS (50 mM NaH_2_PO_4_·2H_2_O and 100 mM NaCl, pH adjusted to 7.2), potential scan rate −5 mV/s.

At first, we have performed the analysis to demonstrate the performance of the designed platinum electrode with AuNPs in WBS without/with H_2_O_2_. It is well known that platinum is a good catalyst for hydrogen peroxide oxidation,^32^ however, the oxidation proceeds at relatively high electrochemical potential. For example, bulk Pt sensors for hydrogen peroxide typically operate at 600–650 mV and therefore could cause significant interference because of the oxidation of other electroactive compounds. Cyclic voltammograms (CVs) were recorded for the bulk Pt electrode without/with the addition of hydrogen peroxide in an electrochemical potential range of 0–400 mV (Figure 1A). When hydrogen peroxide was not added, the electrode potential–current curve did not show significant differences.

After the addition of 1.0 mM H_2_O_2_, the anodic current started to increase from around 200 mV vs Ag/AgCl, but the increase was not significant; that is, at 400 mV the observed current was around 0.9 μA. Afterward, we have tested the performance of a platinum electrode modified with AuNPs (Pt/AuNPs electrode). AuNPs were used because of their catalytic properties toward H_2_O_2_ oxidation,^33^ high catalytic activity comparable to enzymes,^34^ and capacitive properties.^35^ At first, we tested the Pt/AuNP electrode in WBS without H_2_O_2_. CV analysis in the potential range of 0–400 mV demonstrated different characteristics compared to the Pt electrode (Figure 1B). We observed a significant increase in capacitive current, indicating that the Pt/AuNP layer was able to store electric charge. Furthermore, typical reductive currents were observed at potentials lower than 150 mV because of the oxygen reduction reaction.^36^ After adding 1.0 mM H_2_O_2_, the anodic current started to increase from around 180 mV vs Ag/AgCl to around 2.3 μA at 400 mV. This value was significantly higher compared to the unmodified Pt electrode, indicating that the Pt/AuNP electrode had significantly improved properties for H_2_O_2_ oxidation. It was also important to determine the capacitance of the electrodes because we were designing a biosensor based on a capacitive measurement method to improve the sensitivities. We have measured the capacitance of bulk Pt and Pt/AuNP electrodes using an electronic multimeter. The bulk capacitance of the Pt electrode was 1.3 ± 0.05 μF (*n* = 3) while the capacitance of the Pt/AuNP electrode was 8.1 ± 0.3 μF (*n* = 3). A significantly higher capacitance value showed that the Pt/AuNP electrode stores electric charge in addition to performing the catalytic oxidation of H_2_O_2_.

### Capacitor-Based Measurement Method for the Determination of H_2_O_2_

**Figure 2:**
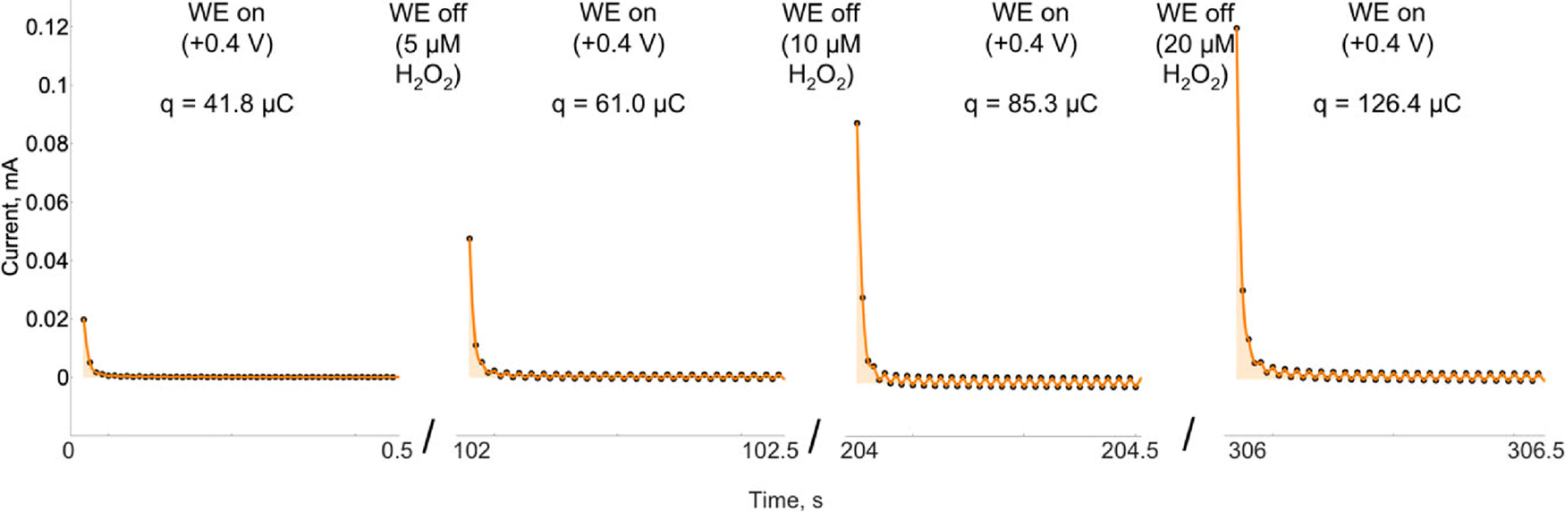
Time–current curve of the Pt/AuNP electrode used to measure the capacitance after the addition of the analyte (H_2_O_2_). During the polarization (WE on), the electrode was kept at 400 mV vs Ag/AgCl to discharge the AuNP layer. During the depolarization stage (WE off), H_2_O_2_ was added and oxidized on the electrode, transferring the charge to the Pt/AuNP electrode. The black points demonstrate the measurement points, the orange curves – the interpolating curves, and the highlighted area in orange shows the accumulated electric charge. WBS (50 mM NaH_2_PO_4_·2H_2_O and 100 mM NaCl, pH adjusted to 7.2). The slight oscillations visible in the graphs appeared when magnetic stirring of the solution was applied.

After demonstrating that the Pt/AuNP electrode was capable of oxidizing H_2_O_2_ at a potential higher than 150 mV and storing electric charge, we have applied a capacitance-based measurement method to achieve higher sensitivity and a lower LOD for the intermediate compound H_2_O_2_. Having a high sensitivity and a low LOD for H_2_O_2_ is crucial because amino acid detection is achieved by measuring the relatively low concentration of H_2_O_2_ formed in the enzymatic reaction. The method was based on the catalytic and capacitive properties of AuNPs. Basically, the measurement algorithm was created following the steps below. At first, the electrode was polarized at 400 mV for 200 s to fully discharge the Pt/AuNP layer. Subsequently, the Pt/AuNP electrode was disconnected from the circuit, and the appropriate amount of H_2_O_2_ was added. Because the electrode was polarized at 400 mV, AuNPs started to oxidize H_2_O_2_, storing the electrons within the Pt/AuNP layer and, in turn, charging the electrode. Finally, after 100 s, the electrode was connected again to the circuit and polarized to 400 mV, measuring the flowing current during the first 2000 ms. We expected that using this method, very low concentrations of H_2_O_2_ could be detected, because the electrode stores the electrons received from H_2_O_2_ and the total signal value could be accumulated and amplified using the time given to charge the electrode. The measurement method was tested using 0, 5, 10, and 20 μM of H_2_O_2_ (Figure 2). At first, the Pt/AuNP electrode was polarized at 400 mV without H_2_O_2_ (Figure 2). The spike in anodic current was measured for 2000 ms, and a total electric charge stored on the electrode was calculated to be 41.8 ± 0.5 μC (shown in orange in Figure 2, ∼0 s). This value was the blank capacitance of the Pt/AuNP electrode and was related to the capacitive currents of AuNPs. Furthermore, the blank capacitance values were reproducible, and multiple measurements gave similar blank capacitance values with a low standard deviation (1.10 μC). After polarization, the Pt/AuNP electrode was disconnected from the circuit (WE off), and 5.0 μM H_2_O_2_ was added. The solution was mixed for the first 10 s, allowing H_2_O_2_ to be oxidized by the polarized electrode, accumulating the charge. The electrode was again polarized connecting to the circuit to the potential at 400 mV and measuring the current spike for 2000 ms (Figure 2, ∼102 s). The resulting charge was calculated to be 61 ± 4 μC, demonstrating that the peroxide was oxidized by the electrode and the charge accumulated by the AuNPs. We have carried out additional measurements adding 10 and 20 μM H_2_O_2_. The resulting current spikes were measured, and charges were calculated 85 ± 2 and 126 ± 8 μC, respectively.

**Figure 3:**
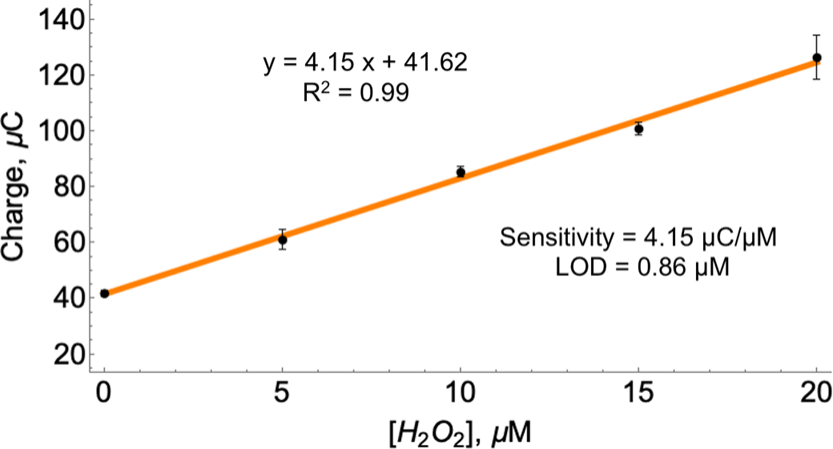
Calibration curve of the Pt/AuNP electrode using the charge capacitive method for the detection of H_2_O_2_ in the range of 0–20 μM. A typical time–current curve used to calculate the capacitance values is shown in Figure 2. Measurements to produce error = 3. WBS (50 mM NaH_2_PO_4_·2H_2_O and 100 mM NaCl, pH adjusted to 7.2).

Because capacitive time–current measurements demonstrated that the charge of the Pt/AuNP electrode depended on the concentration of H_2_O_2_, a calibration was performed using H_2_O_2_ in the range of 0–20 μM (Figure 3). The calibration curve demonstrated that electrode charge on concentration followed a linear dependence in the investigated range. Analytical parameters such as sensitivity and LOD were calculated to be 4.15 μC/μM and 0.86 μM, respectively. The parameters received demonstrated that the designed Pt/AuNP electrode in conjunction with the capacitive measurement method used showed good analytical parameters for H_2_O_2_ detection and can be further used to apply the enzymatic membrane for the analysis of total L-amino acids.

### Electrochemical Analysis of the Pt/AuNP/Enz Electrode and Calibration of the Biosensor

**Figure 4:**
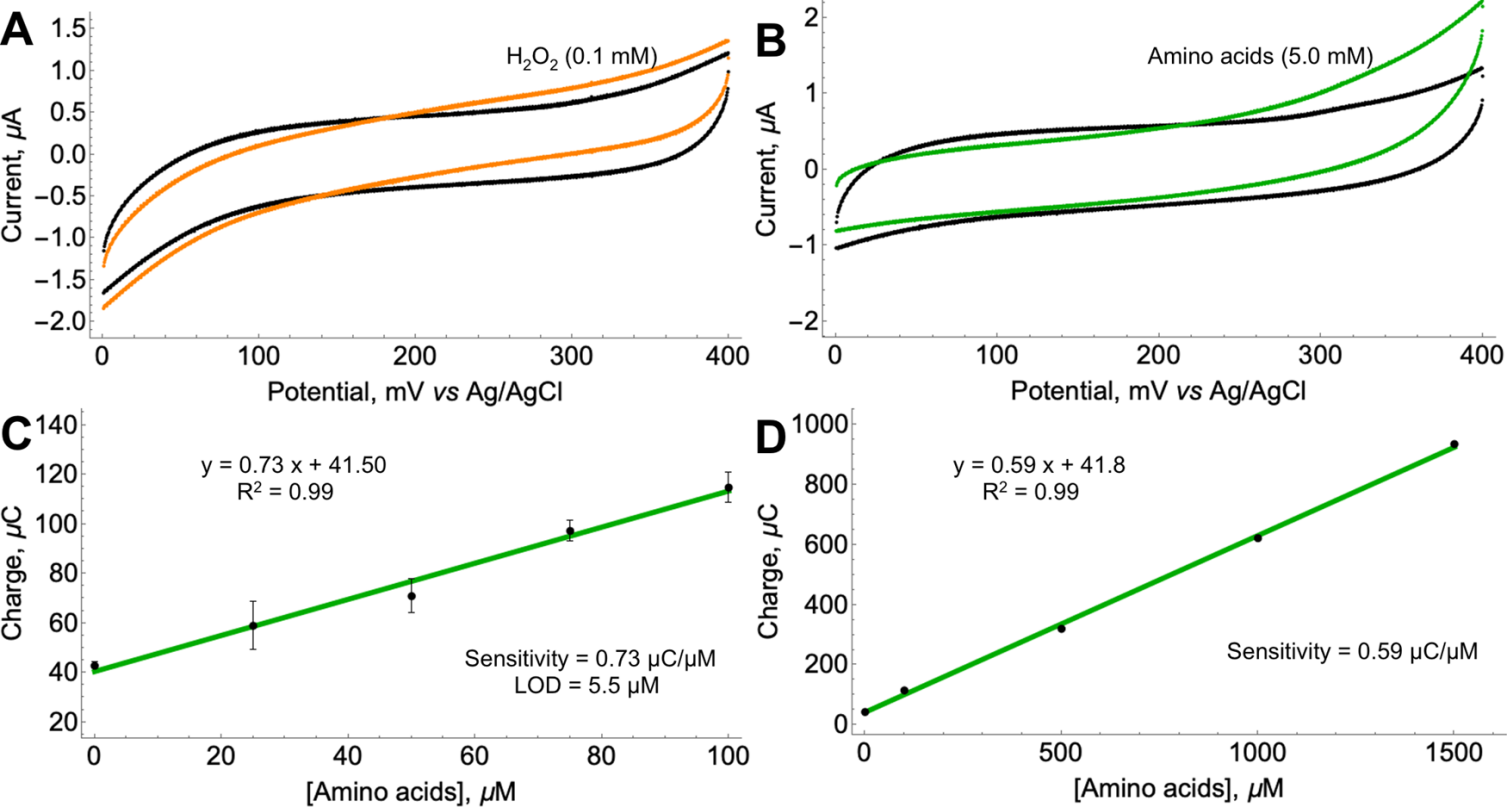
Electrochemical analysis and calibration of Pt/AuNP/Enz electrodes. (A) CV of the electrode without (black) and with (orange) 0.1 mM H_2_O_2_. Potential scan rate −5 mV/s. (B) CV of the electrode without (black) and with (green) 5.0 mM L-amino acids. Potential scan rate −5 mV/s. (C) Calibration curve of the Pt/AuNP/Enz electrode using the charge capacitance-based method for the detection of amino acids in the range of 0–100 μM. Time–current curves used to calculate capacitance values are given in Supporting Information, Figure S3. (D) Calibration curve of the Pt/AuNP/Enz electrode using the charge capacitive method for L-amino acids in the range of 0–1500 μM. Time–current curves used to calculate capacitance values are given in Supporting Information, Figure S5. Measurements to produce error = 3. WBS (50 mM NaH_2_PO_4_·2H_2_O and 100 mM NaCl, pH adjusted to 7.2).

After demonstrating that the Pt/AuNP electrode was capable of oxidizing H_2_O_2_ and storing electric charge in the AuNP, we modified the electrode with the enzyme membrane for the oxidation of L-amino acids. The membrane was composed of immobilized L-amino acid oxidase (LAOx) on a flexible support made from PET using albumin and glutaraldehyde. Because of the broad and unspecific activity of LAOx in the oxidation of amino acids,^37^ the membrane was capable of oxidizing most L-amino acids near the surface of the electrode, in turn producing H_2_O_2_. The final iteration electrode with the LAOx membrane used for L-amino acid analysis was named Pt/AuNP/Enz. At first, we have shown that the electrode was still capable of oxidizing H_2_O_2_; that is, the enzymatic membrane did not inactivate the Pt/AuNP layer. CVs without and with 0.1 mM H_2_O_2_ were recorded and revealed that Pt/AuNP/Enz electrodes oxidized H_2_O_2_ (Figure 4A). The increase in anodic current started from around 180 mV and was similar to that of the electrode without the enzymatic membrane. Subsequently, CVs were recorded using the Pt/AuNP/Enz electrode with an analytical standard of L-amino acids, which contains 17 common L-amino acids, each with a concentration of 2.5 mM (except L-cystine at 1.25 mM), typically used to calibrate amino acid analyzers (Figure 4B). The CV was first registered without the analyte and showed no significant electrochemical process. Another CV was recorded after the addition of 5.0 mM L-amino acid standard (concentration sum of all L-amino acids in a standard) and demonstrated a significant increase in anodic current very similar to CVs when H_2_O_2_ was used. Thus, we have shown that Pt/AuNP/Enz electrodes were capable of oxidizing both H_2_O_2_ and L-amino acids.

The concentration of L-amino acids in real samples, accounting for the typical sample dilution, is expected to be low, most likely in the range of a few hundred of micromoles per liter; thus most likely conventional constant potential amperometry is not suitable for the analysis. Therefore, to calibrate the Pt/AuNP/Enz electrode and receive the biosensor, we used the capacitance-based method as described above. The Pt/AuNP/Enz electrode was placed in a cell and polarized at 400 mV without L-amino acids. The anodic current spike was measured for 2000 ms, and the total electric charge stored in the electrode was calculated to be 42 ± 1.2 μC. This value was the blank capacitance of the Pt/AuNP/Enz electrode and was related to the capacitive currents of AuNPs. Control measurements using different measurement sequences (before/after addition of L-amino acids) demonstrated that the blank capacitance of Pt/AuNP/Enz electrodes was reproducible (Supporting Information, Figure S1) and similar to a blank electrode charge without the enzymatic membrane (41.8 ± 0.5 μC). After electrode polarization, the Pt/AuNP/Enz electrode was disconnected from the circuit, and 25 μM L-amino acids were added into a cell. The solution was mixed for 100 s when the electrode was disconnected (WE off time), allowing the enzymatic membrane to oxidize L-amino acids forming H_2_O_2_ and H_2_O_2_ to be oxidized by the polarized electrode, accumulating the charge. Additionally, we investigated the duration of WE off time when analyzing Pt/AuNP/Enz electrodes, due to the influence on sensitivity (Supporting Information, Figure S2). The results demonstrated that WE off time shorter than 100 s significantly decreased the electrode sensitivity, while longer time increased the sensitivity but also made the measurements unreasonably longer. For those reasons, to further analyze the electrodes, we used the WE off time of 100 s. The electrode was polarized again, connecting it to the circuit, and current spikes were measured. The resulting capacitance was calculated to be 59 ± 9 μC, demonstrating that the Pt/AuNP/Enz electrode operated on the same principle as the Pt/AuNP electrodes. The Pt/AuNP/Enz electrodes were calibrated in the concentration range 0–100 μM (Figure 4C and Supporting Information, Figure S3). Analytical parameters (sensitivity and LOD) in the low-concentration range were estimated to be 0.73 μC/μM and 5.5 μM, respectively. For comparison, we also calibrate the electrodes using a conventional constant potential amperometry with L-amino acid standard in the range of 0–100 μM (Supporting Information, Figure S4). The results received demonstrated that the amperometric method was not suitable for the analysis because of the lack of sensitivity—the current increase after the addition of L-amino acids was barely recognizable from the background noise and was also very hard to reproduce. The capacitive method allowed us to expand the calibration range to measure at significantly higher concentrations using the same method. In Figure 4D, we have shown that the Pt/AuNP/Enz electrode could also be applied to measure L-amino acids in a concentration range 0–1500 μM (the time–current curve used to calculate the capacitance values is given in Supporting Information, Figure S5). Control measurements in which an AuNP-unmodified Pt/Enz electrode was calibrated with L-amino acids using the capacitance-based method also demonstrated a capacitance increase after the addition of analytes (Supporting Information, Figure S6) because of electrode ability to store the same charge. However, the analytical parameters were significantly lower (sensitivity and LOD were 0.38 μC/μM and 20.1 μM, respectively) indicating the advantage in the use of AuNPs. The stability of the Pt/AuNP/Enz electrode during storage was also investigated (Supporting Information, Figure S7). Data have shown that the Pt/AuNP/Enz electrode has an adequate stability and retained around 50% of the initial activity after 10 days of storage.

### Analysis of L-Amino Acid Loss in Human Serum Samples for Patients Undergoing Renal Replacement Therapy

**Figure 5:**
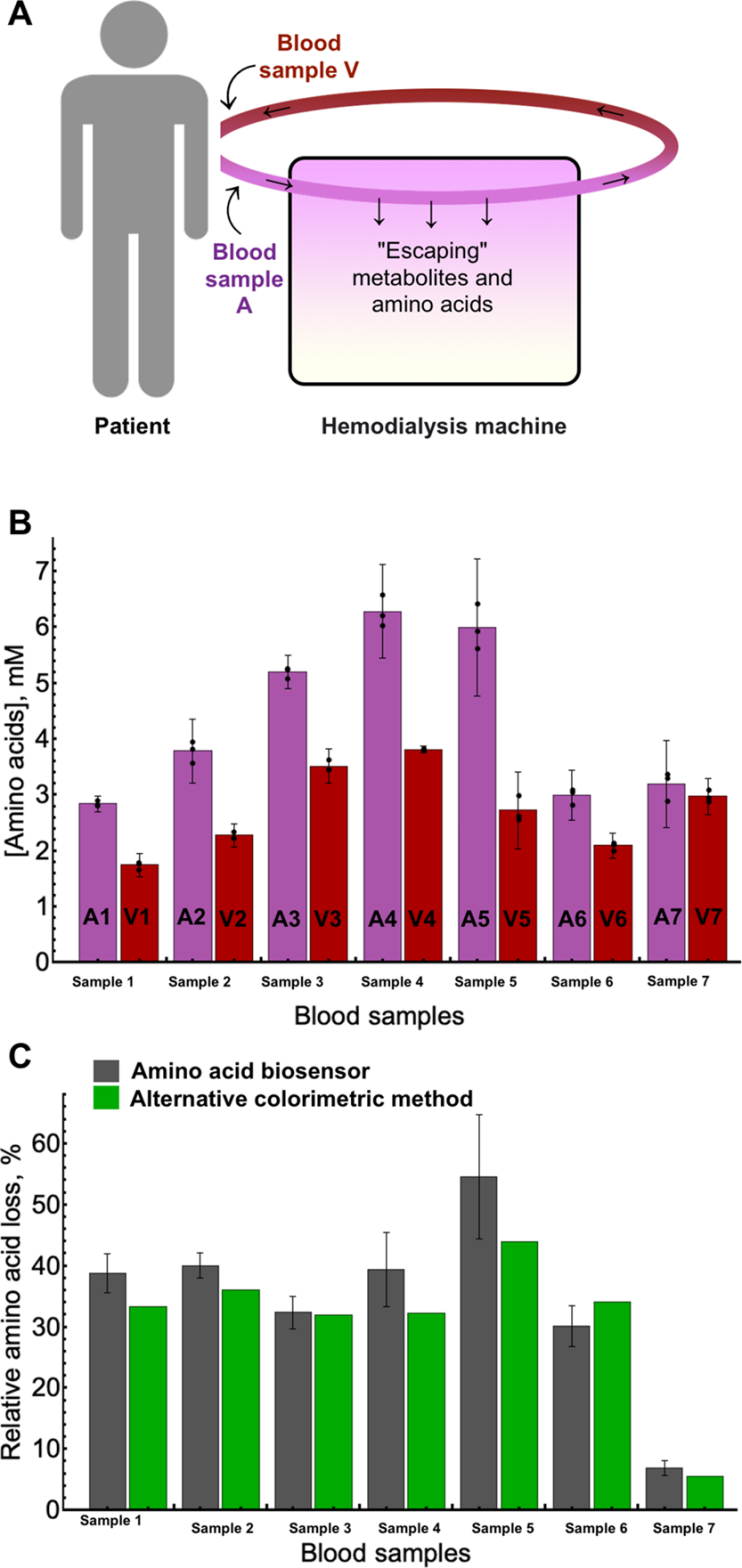
Analysis of serum samples. (A) Scheme demonstrating the origin of the serum samples analyzed. Two samples of the same patient were analyzed: serum obtained from blood before entering the hemodialysis apparatus (Sample A) and serum obtained from blood leaving the hemodialysis apparatus (Sample V). (B) Sample analysis with the Pt/AuNP/Enz electrode (absolute values). (C) Sample analysis with a Pt/AuNP/Enz electrode and an alternative colorimetric kit (relative values, that is, amino acid loss, %).

We have applied the designed electrode for measuring the real samples: human serum received from blood from hospital patients undergoing renal replacement therapy. The patients gave their consent to participate in biomedical research, and the project was approved by a Vilnius Regional Biomedical Research Ethics Committee (Lithuania), approval number: 2021/2-1306-784. Blood samples from which serum was received and analyzed using biosensors were taken from patients, as demonstrated in Figure 5A, that is, two samples were taken and analyzed for each patient. The first sample was taken from the blood before entering the hemodialysis apparatus (Figure 5A, Blood sample A), while the second blood sample was taken from the blood leaving the apparatus (Figure 5A, Blood sample V).

Clinically, it is important to analyze and compare the level of amino acids between samples A and V, because hemodialysis apparatus is expected to ’wash out’ not only toxic metabolites but also amino acids.^38^ Thus, these measurements could provide the answer to the question: what is the percentage of amino acids lost after the hemodialysis cycle?

We applied the designed Pt/AuNP/Enz electrode using a capacitance-based method to analyze the serum samples and compared the sensor response with the alternative colorimetric method for total L-amino acid analysis. Absolute amino acid concentrations measured with the Pt/AuNP/Enz electrode in conjunction with the capacitance-based measurement method are given in Figure 5B and Table 1. Measurements showed that for all samples analyzed, the biosensor measured amino acid loss, that is, all serum samples A had a lower level of amino acids compared to samples V. Furthermore, the total initial levels of L-amino acids in the serum were comparable from patient to patient (1.74–6.28 mM).

**Table 1:** Amino Acid Concentration Measured in Human Serum Samples with the Developed Biosensor (the Pt/AuNP/Enz Electrode) and the Alternative Colorimetric Method

sample	amino acids according to the biosensor (*n* = 3), mM	relative loss according to the biosensor, (*n* = 3), %	amino acids according to the colorimetric method, mM	relative loss according to the colorimetric method, %	difference between two methods, %
A1	2.84 ± 0.14	38.7 ± 3.2	0.95	33.3	5.4
V1	1.74 ± 0.2		0.63		
A2	3.78 ± 0.57	40.0 ± 2.1	1.45	34.2	5.8
V2	2.27 ± 0.2		0.96		
A3	5.19 ± 0.3	32.3 ± 2.7	1.08	31.9	0.4
V3	3.51 ± 0.3		0.73		
A4	6.28 ± 0.84	39.3 ± 6.1	1.38	32.2	7.1
V4	3.80 ± 0.06		0.94		
A5	5.99 ± 1.2	54.5 ± 10.2	2.08	42.9	11.6
V5	2.72 ± 0.7		1.19		
A6	3.00 ± 0.44	30.1 ± 3.3	1.56	34	3.8
V6	2.09 ± 0.22		1.03		
A7	3.2 ± 0.8	6.8 ± 1.2	2.10	5.5	1.3
V7	2.97 ± 0.3		1.98		

When comparing the absolute amino acid concentrations measured using the Pt/AuNP/Enz biosensor with the alternative colorimetric method, we observed significant differences (Table 1). However, differences in absolute concentration values were expected—measurement of L-amino acids using a colorimetric kit took around 1 h, and the principle was to oxidize all the amino acids present in a particular volume. In contrast, the measurement with the Pt/AuNP/Enz electrode took less than 2 min, and the absolute result was closely related to the specificity of the L-amino acid oxidase. This means that high-activity amino acids would contribute more to the signal in comparison to low-activity amino acids, thus leading to differences between methods in measuring the total amino acid concentration. Because the clinically important parameter was the relative loss of amino acids, that is, the comparison of samples A with samples V, we analyzed the concentration changes measured by the Pt/AuNP/Enz biosensor and the alternative colorimetric method (Figure 5C). In this case, both methods gave a solid agreement (Table 1)—the Pt/AuNP/Enz electrodes and colorimetric kit gave a difference between the samples in the range of 0.4–11.6%. Thus, to summarize, the designed Pt/AuNP/Enz electrode was successfully applied to measure amino acid loss in diluted serum samples received from blood from patients undergoing renal replacement therapy and was especially applicable and accurate once the relative amino acid loss was measured by comparing the amino acid level from samples before and after the hemodialysis apparatus.

## Conclusions

In this paper, we present a biosensor for the detection and quantification of amino acids based on an AuNP-modified Pt electrode with an adjusted membrane containing crosslinked L-amino acid oxidase. The Pt/AuNP layer had both catalytic and capacitive properties: it was able to oxidize hydrogen peroxide and store the received electric charge in the AuNPs. To measure the concentrations, we used a capacitance-based method. The electrode was polarized at 400 mV (vs Ag/AgCl) to fully discharge the Pt/AuNP layer and disconnected from the circuit, and the appropriate amount of analyte was added. Because the electrode was polarized at 400 mV, AuNPs started to oxidize H_2_O_2_, storing the electrons within the Pt/AuNP layer and in turn charging the electrode. The electrode was then connected to the circuit and polarized again to 400 mV measuring the flowing current and calculating the electric charge stored in an AuNP layer. The described method allowed for a reproducible and sensitive detection of H_2_O_2_ with a sensitivity and an LOD of 4.15 μC/μM and 0.86 μM, respectively. Afterward, we have designed and tested the electrode for L-amino acid measurements using the Pt/AuNP electrode as a base with an additional enzymatic membrane composed of cross-linked L-amino acid oxidase (Pt/AuNP/Enz electrode). The Pt/AuNP/Enz electrodes in conjunction with the developed capacitive method were applied to measure amino acids in low (0–100 μM) and high (0–1500 μM) concentration range. Analytical parameters, that is, sensitivity and LOD (0.73 μC/μM and 5.5 μM, respectively) were received for L-amino acids, indicating that the developed electrode is one of the most sensitive biosensors for the measurements of L-amino acids. Finally, the designed biosensor was applied to measure relative loss of L-amino acids for hospital patients undergoing renal replacement therapy by comparing amino acid levels in diluted serum samples received from blood before/after entered/exiting the hemodialysis apparatus and demonstrated fast assessment and good agreement with the alternative colorimetric method.

## Supporting Information Available

Control experiments, measurements of electrode sensitivity dependence of charging time, electrode stability data, additional calibration curves, and a comparison between the proposed capacitance-based method and conventional constant potential amperometry (PDF)
