# Supplementary material for: Capacitance-Based Biosensor for the Measurement of Total Loss of L-Amino Acids in Human Serum during Hemodialysis
Source: ACS Sens. 2022 Oct 21;7(11):3352–9. doi: 10.1021/acssensors.2c01342 (PMC9706805; doi:10.1021/acssensors.2c01342)
Supplement: Supplementary file 1 — se2c01342_si_001.pdf [file se2c01342_si_001.pdf]

Capacitance-based Biosensor for the Measurement of Total Loss of L-amino acids in Human Serum During Hemodialysis

Justas Miškinis,[a] Eimantas Ramonas,[a] Vidutė Gurevičienė,[a] Julija Razumienė,[a] Marius Dagys, and Dalius Ratautas [a]*

[a] Life Science Center, Vilnius University, Saulėtekio al. 7, LT-10257 Vilnius, Lithuania

* Corresponding author Dalius.Ratautas@gmc.vu.lt

Supporting Information

Supporting Figures

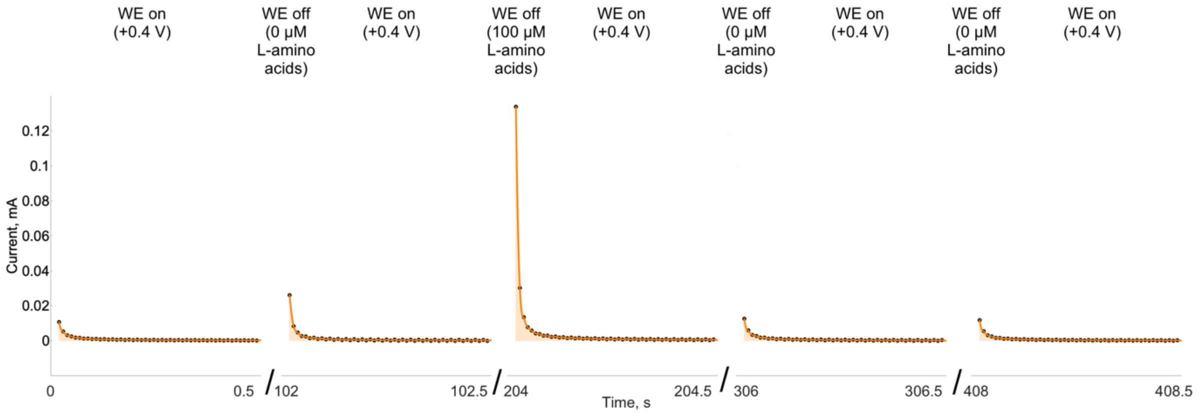

Figure S1. Control measurements demonstrating Pt/AuNP/Enz biosensor blank capacitance without L-amino acids (0 µM) before (peaks 1 and 2) and after (peaks 4 and 5) the addition of 100 µM L-amino acids.

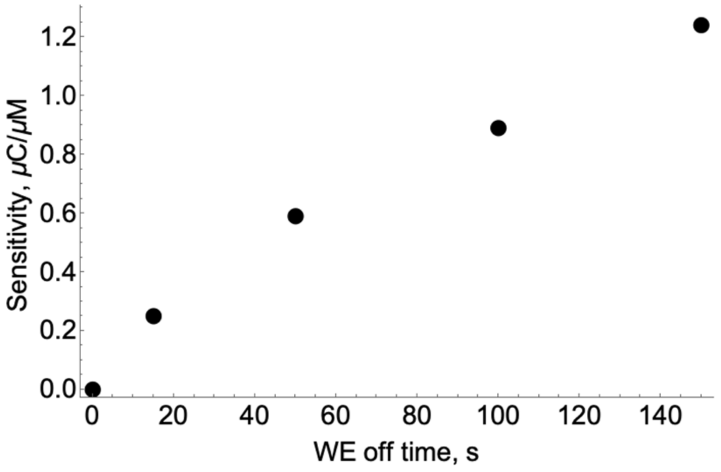

Figure S2. Sensitivity dependence of Pt/AuNP/Enz electrodes on WE off (charging) time. Data indicate that increase in charging time results in increase in the sensitivity. However, the increase in the sensitivity after 100 s was not merit the time, i.e., the measurements became unreasonably too long. The sensitivity at 100 s was sufficient for analysis of samples.

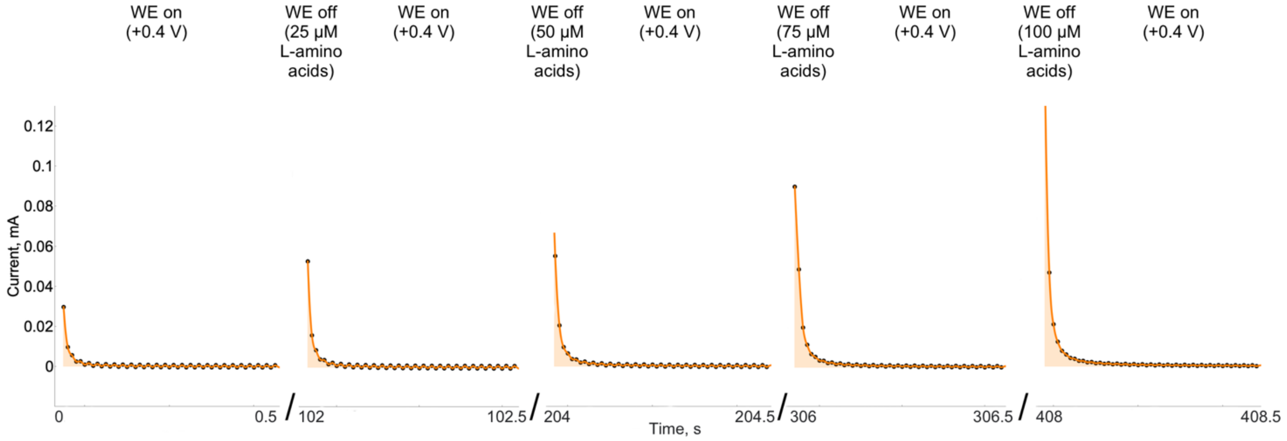

Figure S3. A typical time-current curve demonstrating Pt/AuNP/Enz biosensor calibration using L-amino acid standard in low concentratation range (0–100 µM). The area highlighted in orange shows the current used to calculate the capacitance values.

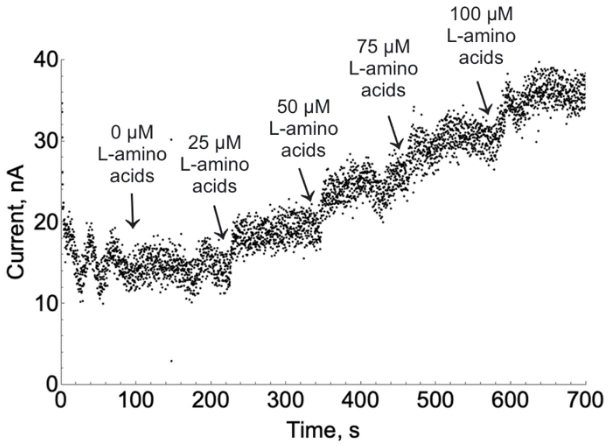

Figure S4. Pt/AuNP/Enz biosensor calibration with low L-amino acid standard concentrations (0–100 µM) using conventional constant potential amperometry. The given curve demonstrates that conventional constant potential amperometry is not a well suitable method for electrode analysis. The signal is barely recognizable from the noise and is difficult to reproduce.

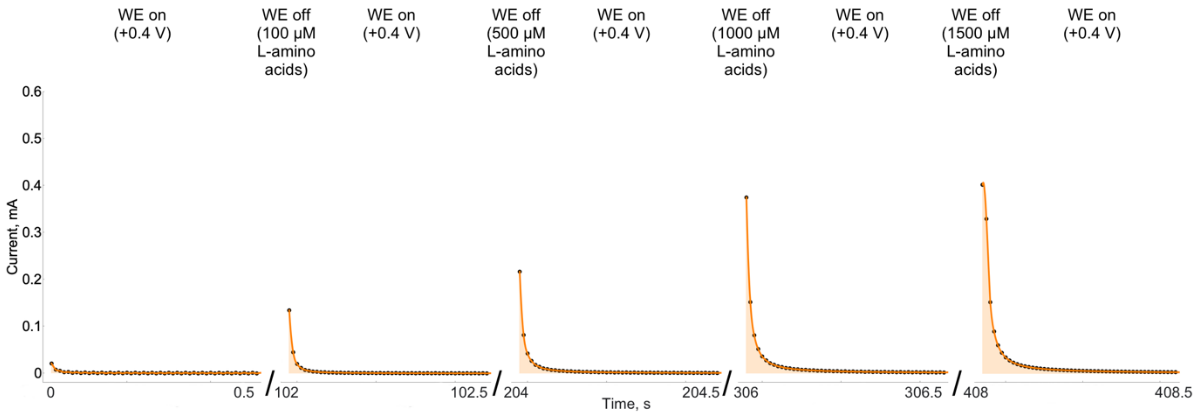

Figure S5. A typical time-current curve demonstrating Pt/AuNP/Enz biosensor calibration using L-amino acid standard in high concentration range (0–1500 µM). The area highlighted in orange shows the current used to calculate the capacitance values.

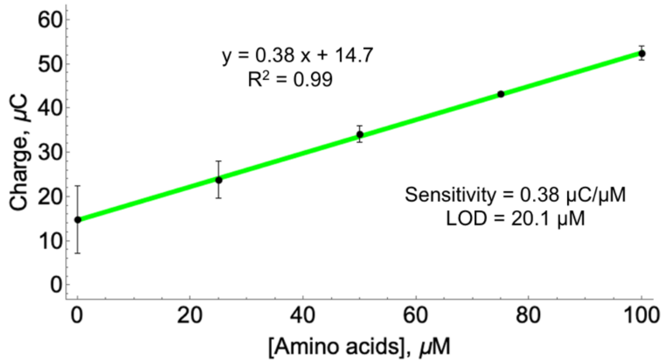

Figure S6. Control calibration curve of the Pt/Enz electrode using charge capacitance-based method for detection of amino acids in the range 0–100 µM. The curve demonstrates that removal of AuNPs reduces the electrode performance since the analytical parameters were significantly lower in comparison to Pt/AuNP/Enz biosensors (sensitivity and LOD were 0.38 vs 0.73 µC/µM and 20.1 vs 5.5 µM, respectively) indicating the advantage of in AuNPs usage.

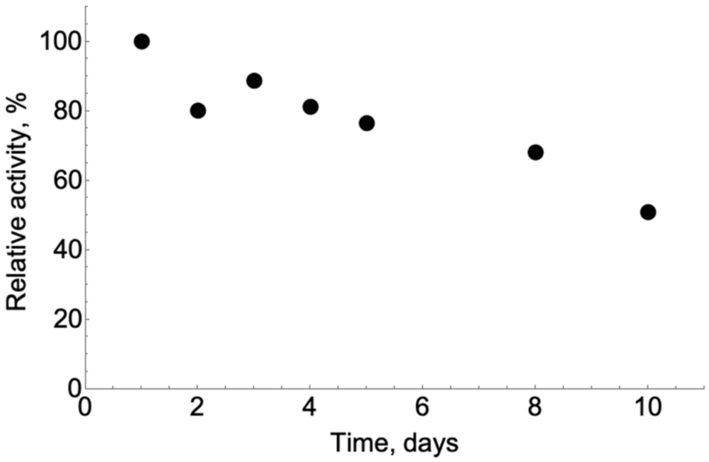

Figure S7. Relative activity dependence of Pt/AuNP/Enz electrodes storage time.
